# Social practice, plural lifestyles and health inequalities in the United Kingdom

**DOI:** 10.1111/1467-9566.12780

**Published:** 2018-08-13

**Authors:** Jens Kandt

**Affiliations:** ^1^ The Bartlett Centre for Advanced Spatial Analysis University College London London UK

**Keywords:** health inequalities, social practice, lifestyles, health‐related behaviours, cluster analysis

## Abstract

Persistent health inequalities pose a continued research and policy challenge in the United Kingdom and elsewhere. Current approaches to health research and promotion are predicated on a distinction between wider, social structural causes and individual, health‐related behaviours often conceived of as lifestyle choices. Drawing on Bourdieu's theory of social practice, this paper develops an integrated perspective by observing associations between health and structured lifestyle practices. Using the UK Understanding Society household survey, a taxonomy of eight lifestyle clusters is identified, which exhibit significant health inequalities on a number of indicators. But the plurality of practices and subjective orientations inherent in the taxonomy reveals a finer, more complex differentiation of the social gradient in health. In addition, lifestyle appears to at least in parts mediate the relationship between social, material conditions and health. A feature of the taxonomy is that it admits a relational and contextual apprehension of health‐relevant, behavioural aspects within a more holistic notion of lifestyles. Based on this view, strategic approaches can be developed that respond to group‐specific situations and pathways and their varying roots in upstream or downstream domains of policy.

## Introduction

Despite unprecedented attempts to address health inequalities strategically in the United Kingdom, inequalities continue to widen, and only little progress has been observed (Bambra 2012, Mackenbach 2011, Scambler and Scambler 2015). A frequently reiterated explanation for the UK programme's failure is that policy approaches ‘drifted’ (Popay *et al*. 2010) into downstream interventions, unduly focusing on individual behaviours instead of wider structural determinants of health disadvantage and risk (Baum and Fisher 2014, Garthwaite *et al*. 2016). This is a feature of not just the UK programme, the World Health Organisation has recently ascertained that lifestyle risk factors pose a significant threat to human health as non‐communicable diseases ascend to the leading cause of death worldwide (WHO 2014). The organisation emphasises at various places in their 2014 report the need to design policy interventions that influence behaviour with respect to smoking, drinking, unhealthy eating and physical inactivity. The fundamental assumption is that these habits are primarily guided by free will and are hence modifiable.

This rationalistic notion of behaviour has not only been criticised for general epistemological limitations (Byrne 2002, Forbes and Wainwright 2001, Wainwright and Forbes 2000) but also for an exaggeration of the role of individual choice over structural conditions (e.g. Abel and Frohlich 2012, Frohlich *et al*. 2001, Garthwaite *et al*. 2016, Williams 1995). Health‐related behaviours are in fact embedded in conscious and unconscious everyday life routines, which shape and are shaped by the practical reality of social structure (Baum and Fisher 2014, Cockerham *et al*. 1997, Williams 1995). By contrast, a rationalist view artificially dichotomises agency and structure and prevents a relational understanding of behaviour as social practice (Veenstra and Burnett 2014a, 2014b).

Researchers have thus called for alternative approaches to theorise, conceptualise and study health inequalities, wherein health‐related behaviours are conceived of as expressions of practical tendencies formed within relatively stable ‘behavioural’ routines and social milieus shaped by prevailing relations of power (Byrne 2002, Veenstra and Burnett 2014b, Scambler 2007). Yet, at least among quantitative studies, there is but sparse experience with empirical applications of such alternative approaches.

The objective of this paper is to address this gap and employ an approach that engages a relational and contextual notion of practice informed by Bourdieu's social practice theory (Bourdieu 1977, 1984) and more recent, sociological debates on class in general (Bennett *et al*. 2009, Savage *et al*. 2013) and health in particular (Abel and Frohlich 2012, Veenstra and Burnett 2014a, 2014b). The paper will show how health inequalities can be viewed more holistically within a differentiated perspective of lifestyles and discuss how this may inform health research and promotion.

## Conceptual approach: from individual behaviour to health practices

Bourdieu's theory of social practice has regained attention in the inquiry into the causes of persisting health inequalities (e.g. Abel and Frohlich 2012, Jones and Williams 2017, Veenstra and Burnett 2014a). One of the most salient ideas taken from Bourdieu is, in brief, that individuals’ practical social experience over time fashions dispositions and stable tendencies to act and react, structuring subjective perceptions of behavioural possibilities and impossibilities (Bourdieu 1984). Health‐relevant behaviours, too, are expressions of these tendencies, constituted by habitus, a mental ‘structuring structure, which organizes practices and the perception of practices’ (Bourdieu 1984: 170). They are deeply embedded in daily life and follow their own situational, ‘practical logic’ (Bourdieu 1990: 90), which typically differs from an abstract, rationalist logic employed by outside observers of these actions (Bourdieu 1977).

The interplay of dispositions (habitus), position in social space and the time and culture‐specific ‘position‐takings’ finds expression in taste, values and subjective judgements, all of which inhere in lifestyle choices in diverse domains of practice, including food, sports, arts consumption or politics. The structuring structure of habitus makes these domains homologous; according to Bourdieu, an agent's relations in social space – shaped by the relative possession of economic, cultural and social capital – translate into relations between lifestyle practices: ‘The habitus is this generative and unifying principle which retranslates the intrinsic and relational characteristics of a position into a unitary lifestyle, that is, a unitary set of choices, persons, goods, practices’. (Bourdieu 1998: 8).

Class‐affiliated taste and dispositions result in the differentiation of lifestyles rendering social stratification tangible in everyday life: ‘The space of objective differences (with regard to economic and cultural capital) finds an expression in a symbolic space of visible distinctions, of distinctive signs which are so many symbols of distinction’. (Bourdieu 1987: 11). This congruent or homologous relationship between social space and the space of lifestyles implies that agents located more closely in social space of objective differences find themselves closely located in the symbolic space of lifestyles and, in theory, are more likely to encounter each other and partake in similar social experience in everyday life. Health researchers conclude that there is a plurality of motivations and orientations that, being derived from an agent's relative position in social space, drive health‐related practices (Veenstra and Burnett 2014b, Williams 1995).

Indeed, health studies employing Bourdieu's lifestyle concepts find homologous relations between formal social status categories, lifestyles and health. Veenstra (2007), for example, applied Bourdieu's preferred statistical method, Multiple correspondence analysis (MCA), and identified the social and lifestyle contexts in which certain health outcomes and health‐related practices can be observed. In his study of a community in British Columbia, Canada, he uncovered a social space of lifestyle practices defined by volume of economic and educational capital and social integration, thus revealing the relational context of health‐related behaviours. Similar insight is offered by Gatrell *et al*. (2004) in selected communities in the United Kingdom.

Yet, in a parallel debate on social class, some critics question the definite force of economic and cultural capital in producing class habitus and thus shaping lifestyle practices (Bennett *et al*. 2009). In particular, the MCA‐based joint descriptions of social and symbolic space tend to emphasise relations between the most distinguishing practices at the expense of practices shared across different social contexts (Bennett *et al*. 2009). By associating particular lifestyle choices with specific configurations of capital, finer differentiations, formed along other patterns of taste remain concealed, which results in an unrealistic picture of unity and coherence. Habitus is likely shaped by factors of more complex social provenance, which often escape measures of formal social categories. From a health perspective, it may be added that the effect of practices adopted across social classes are likely to be as health‐relevant as the most distinguishing practices. Smoking, for example, affects lung functioning independently from the discriminating force of this practice.

Abel (1991) developed an alternative approach potentially addressing these limitations. He employed cluster analysis to identify lifestyle groups in the United States and Western Germany based on health‐related behaviours, attitudes and personal values. Although the study did not draw on Bourdieu, the results demonstrate that a lifestyle taxonomy can provide a fuller understanding of health and healthy practices within finer social differentiations that may run across formal categories of class and capitals. To date, studies developing comprehensive lifestyle taxonomies remain rare in health research.

This paper aims to characterise health inequalities within plural lifestyle groups in the United Kingdom by considering health‐related practices alongside activities and subjective orientations on other relevant lifestyle domains. In Bourdieu's terms, lifestyle domains may be conceptualised as fields, which are relatively autonomous spheres of social life where social positioning is asserted and contested through the medium of practices (Bourdieu 1984). Competing practices, their perception and experience may trigger various pathways linking social conditions to health through not only direct bodily impacts but also acquisition of symbolic status and prestige. In the context of fields, practices and capitals become inextricably linked and thus health‐relevant (see Pinxten and Lievens 2014).

The procedure set out in this paper follows and expands the one adopted by Bourdieu (1984), Bennett *et al*. (2009) and Savage *et al*. (2013), who first construct the space of lifestyle practices and subsequently view the distribution of formal social categories (age, education, etc.) within it (active versus illustrative variables in MCA (Bourdieu 1984). Correspondingly, the present study first generates the lifestyle taxonomy and subsequently employs descriptive statistics of social differences including capitals inherent in this taxonomy. The groups are then characterised in relation to each other based on the joint view of lifestyles, capitals and demographics. Finally, health inequalities among these groups are measured on a range of standardised health indicators, and it is assessed, to which extent lifestyle mediates the widely observed associations between formal social capitals and health outcomes.

## Data and methods

The empirical material for this study is the UK Understanding Society Study (ISER 2015), a nationally representative longitudinal lifestyle survey, conducted annually in the UK since 2009. The survey collects data on a range of health‐relevant practices, attitudes and orientations in thematic modules as well as respondents’ socio‐demographics. Since not all relevant modules are covered in each wave, only individuals that were present at both the second and third waves were included (n = 44,178 unweighted, n = 41,356 weighted). The questions of both waves were pooled and, if they existed in both waves, averaged for each respondent. This approach required the use of longitudinal sampling weights (Knies 2014).

Relevant lifestyle practices and subjective orientations were identified from the health inequalities literature and, following Bourdieu (1984) and Bennett *et al*. (2009), were associated with different fields: health and body, socialising, politics and cultural consumption. The fields and dimensions are arranged according to their proximity to bodily functions based on the idea of anatomy applied in Brunner and Marmot's (2006) social determinants of health model. The result of this procedure is a definition of health‐relevant practices, attitudes and orientations with which to characterise lifestyles in practical and subjective terms (Table 1).

**Table 1:** Social dimensions and lifestyle practices ranked by their pathophysiological imminence

field	dimension	practices, e.g.	studies, e.g.
health & body	1 health‐related activities	smoking, nutrition, alcohol consumption, physical activity	Stringhini *et al*. 2010; Blaxter 1990
socialising	2 social integration and support	presence of and interaction with friends, relatives	Carpiano & Fitterer 2014; Kawachi 2010
	3 social cohesion and trust	local support, local safety, local attachment	Carpiano 2006; Subramanian *et al*. 2003
politics	4 political participation	political competence, interest, perceived benefits	Frohlich & Abel 2014; Wallerstein 1992
	5 civic participation	community engagement, voting, volunteering	Bullen & Onyx 1998; Wallerstein 1992
cultural consumption	6 cultural participation	leisure, visit of events, museums	Pinxten & Lievens 2014; Veenstra 2007
	7 communication	news consumption, digital and social media use	McKinley & Wright 2014; Sommerhalder *et al*. 2009

Solutions from cluster analysis can be heavily affected by missing values. In order to strike an optimal balance between reducing missingness and retaining as many cases as possible, the data was restricted in two stages. First, items with more than 15 per cent of missing cases were excluded. In this process, alcohol consumption was excluded, since the survey only holds this information for a subsample of 78 per cent. Second, respondents with missing values on more than eight variables were excluded and missing values among the remaining respondents were replaced by sample modes. The threshold of eight was chosen because it still retained 90 per cent of all cases (39,965 cases, 37,167 cases weighted – more details on the impact of exclusion are shown in Tables S2 and S3 in online Supplemenary Information). Items were re‐coded as set out in Table S1, those related to health‐related behaviours – smoking, health nutrition and physical activity – were combined to simple additive scales following Stringhini *et al*. (2010).

The cluster analysis proceeded in two stages, combining the advantages of hierarchical clustering and iterative procedures of *k* means clustering (Everitt *et al*. 2011). First, Ward Hierarchical Clustering was used to generate multidimensional cluster means for *k* clusters. These cluster means were used as initialisation of the *k* means algorithm, which has the feature of retrospectively correcting cluster assignments and thus producing more homogeneous groups. The solution with the best ratio of between‐cluster and within‐cluster variance (Everitt *et al*. 2011) was selected and the resulting clusters were verbally characterised. Differences of the groups’ mean *z* scores were tested for statistical significance using oneway ANOVA and Tukey post hoc tests (Tukey 1949).

Health inequalities among the groups were measured using the following six physical health and subjective well‐being variables available in the survey: (i) disability – whether a respondent experienced any form of long‐standing illness limiting activities; (ii) presence of chronic conditions – whether the respondent had any of 16 conditions (e.g. asthma, diabetes, heart attack, stroke) diagnosed in the last twelve months; (iii) hospitalisation – whether a respondent was hospitalised in the last twelve months; (iv) self‐rated health – a five‐point Likert scale question ranging from excellent to poor; (v) subjective well‐being according to the psychometric General Health Questionnaire (GHQ‐12) (Goldberg and Williams 1988); and (vi) life satisfaction – a seven‐point Likert scale ranging from completely satisfied to completely dissatisfied. In order to assess health inequalities between groups, age‐and‐sex standardised relative ratios were calculated for each group and compared with each other.

In a next step, the role of lifestyle clusters in translating characteristics of social space into health outcomes was assessed in a series of binary logit models. Testing for mediation is one way of quantitatively exploring how social conditions translate into health inequalities, although it must be remembered that the habitus generating practices is fashioned by the conditions encountered and practices adopted over the life course, with early childhood environment taking a crucial role. Nevertheless, mediation analysis is useful in delivering some statistical evidence of a mediating role of lifestyle.

Since classic difference‐of‐coefficient methods to assess mediation are not valid for logit models (Van der Weele 2016), the procedure adopted here performs variance partition as a way to estimate mediation through changes in effect sizes (see Lindeman *et al*. 1980, Preacher and Kelley 2011). Effect sizes are then compared for each variable in three types of models, estimated for each health outcome: (i) basic models regressing health outcomes on commonly considered socio‐demographics in the literature, notably age, sex and social status; (ii) fully adjusted models, which in addition to variables in basic models include lifestyle as multinomial variable indicating cluster membership; and (iii) substitution models based on age, sex and lifestyle cluster only.

Mediation is estimated in two ways, first, as the difference in variance accounted for of each variable between fully adjusted and basic models and, second, by comparison of the structure of the variance accounted for in substitution and basic models. All models were implemented in a backward step procedure, which selects candidate variables for inclusion only if they contribute to the variance of the models. Age and sex were pre‐defined candidates in all models to satisfy requirements of age and sex adjustment. Confidence intervals for mediation effects were estimated by bootstrap sampling of 10,000 draws. The software R (R Core Team 2015) was used for analysis.

## Results I: the lifestyle clusters

After a series of tests, an eight‐cluster solution appears to best represent the structure of the data. The clusters differ significantly in their practices and degree of participation on all fields, health and body, socialising, politics and cultural consumption. Table 2 summarises group‐wise *z* scores and indicates statistical similarity between groups for each item. Three broad tendencies can be perceived: social disengagement with unhealthy practices, selective engagement with moderately healthier practices and participation with healthy practices.

**Table 2:** The clusters’ practical characteristics expressed in mean z scores

	1: SD‐X	2: SD‐L	3: SD‐P	4: SE‐P	5: SE‐D	6: P‐X	7: P‐CE	8: P‐NL
healthy nutrition (1)	−.705	−.503	.043	−.169	−.119	.584	.549	.227
level of smoking (1)	.740	.293	−.013^*4,5*^	.007^*3,5*^	−.024^*3,4*^	−.191^*8*^	−.326	−.142^*6*^
alcohol consumption (1)*	−.205^*2,3*^	−.218^*1,3*^	−.143^*1,2*^	.063^*8*^	−.074	.341	.274	.110^*4*^
frequency of doing sports (1)	−.627	−.200	−.746	−.270	.439 ^*7,8*^	.582	.394 ^*5,8*^	.426 ^*5,7*^
days walking at least 30 mins (1)	−.172^*4*^	−.094	−.285^*4*^	−.249^*1,3*^	.225^*7*^	.336	.160^*5,8*^	.123^*7*^
number of close friends (2)	−.319	−.403	−.225	−.155	.217	.452	.424	.127
friends on social networks (2)	−.350	.401 ^*8*^	−.773	−.069^*6,7*^	.631	−.088^*4,7*^	−.121^*4,6*^	.397 ^*2*^
local advice obtainable (3)	.393	−1.100	.457	.225	.284	.550	.382	−.957
sense of belonging to n'hood (3)	.260^*4*^	−1.140	.404 ^*7*^	.247^*1*^	.163	.516	.428 ^*3*^	−.857
ability to borrow things in n'hood (3)	.313	−.957	.107	.245	.378	.651	.395	−.695
perceived n'hood safety (3)	−.172	−.235	−.945	.205^*5,7,8*^	.252^*4,7*^	.363	.262^*4,5*^	.187^*4*^
importance of local friends (3)	.318	−1.120	.468	.231	.191	.553	.438	−.941
willingness to improve n'hood (3)	.027	−1.020	−.037	.202	.143	.459	.409	−.236
proportion of local friends (3)	.245^*3*^	−.252	.313 ^*1*^	.028^*6,7*^	.132	−.031^*4,7*^	.020^*4,6*^	−.423
intention to stay in n'hood (3)	.284^*4,7*^	−.896	.421	.250^*1*^	.036	.343 ^*7*^	.315 ^*1,6*^	−.755
regular talking to neighbours (3)	.296	−1.120	.360	.232	.221	.520	.408	−.911
sense of civic duty (4)	−.790	−.619	.293	.340	−.709	.605	.550	.250
perceived political qualification (4)	−.695	−.490 ^*5*^	−.179	.281	−.517 ^*2*^	.640	.527 ^*8*^	.577 ^*7*^
effort of political engagement (4)	.411	.119	.216	−.038	.046	−.282^*7,8*^	−.306 ^*6,8*^	−.322 ^*6,7*^
informed about politics (4)	−.696	−.455 ^*5*^	.187^*6*^	.467	−.474 ^*2*^	.210^*3*^	.072^*8*^	.066^*7*^
interest in politics (4)	−.874	−.605	−.004	.298	−.638	.719	.642	.635
perceived benefit in voting (4)	−.553 ^*5*^	−.390	.374 ^*4,6*^	.386 ^*3,6*^	−.545 ^*1*^	.424 ^*3,4*^	.261	.163
intention to vote (4)	−1.280	−.750	.493 ^*4,7*^	.527 ^*3,7*^	−.685	.572	.509 ^*3,4*^	.411
member of organisation (5)	−.519 ^*2*^	−.479 ^*1*^	−.312	−.250^*5*^	−.217^*4*^	.500	1.500	.032
volunteering (5)	−.306 ^*4,6*^	−.260^*3,4,6*^	−.253^*2*^	−.301 ^*1,2,6*^	−.183	−.292^*1,2,4*^	2.380	−.027
participation in arts activities (6)	−.814	−.268^*3,4*^	−.176^*2,4*^	−.216^*2,3*^	.170	.486 ^*7*^	.484 ^*6*^	.404
attendance of art events (6)	−.916	−.217	−.835	−.311	.408	.625	.561	.637
visits to historic sites (6)	−.760	−.487	−.511	−.337	.097	.843	.742	.605
visits to museums/galleries (6)	−.591	−.430 ^*4*^	−.504 ^*4*^	−.466 ^*2,3*^	−.093	.935	.766	.626
internet use (7)	−.668	.224	−1.640	.383	.477	.311	.296	.526
news consumption (7)	−.610	−.292	−.489	−.039	.038	.532	.450	.465
TV consumption (7)	.515	.108	.767	.023	−.221^*6*^	−.269^*5,7*^	−.318 ^*6*^	−.428
Frequency	10%	12%	13%	13%	15%	14%	9%	14%

**clusters in columns:** Social Disengagement ‐ Extensive (SD‐X), Local (SD‐L), Physical (SD‐P); Selective Engagement ‐ Political (SE‐P), Digital (SE‐D); Participation ‐ Extensive (P‐X), Civically Engaged (P‐CE), Non‐Local (P‐NL) · **notes:** All pairwise differences are statistically significant at p ≤ .05, except those pairs that are indicated in superscript above. Reading example for first row, first column: the score distribution of health nutrition for SD‐X is statistically identical to that of cluster 6 (SE‐D). Scores larger than +/‐ .3 are underlined. *Alcohol consumption not used in clustering.

### Social disengagement and unhealthy practices

Three clusters exhibit social disengagement (SD); they appear more detached and withdrawn from social life than other clusters. The SD‐Extensive is characterised by the absence on most fields (high negative *z* scores), notably collective domains such as politics and extra‐domestic cultural consumption. While TV consumption is high (.515), individuals tend to be less physically active (–.627), consume less healthy nutrition (–.705) and smoke more often (.740). The SD‐Local cluster is less detached from the fields of cultural consumption and politics but indicates extremely low local social integration (most *z* scores smaller than –1). Members of this cluster eat less healthily (–.503) and smoke more often (.293). The third cluster SD‐Physical shows reduced engagement in any form of physical activity, be it exercising (–.746), walking (–.285) or extra‐domestic cultural participation (most ranging from –.504 to –.835). Whereas this cluster does not use digital technology (Internet use –1.64), it shows more engagement with the local social environment.

### Selective engagement and moderately healthier practices

Selective engagement (SE) is engagement on predominantly one or two fields. Two clusters fall into this grouping, and compared with SD clusters, each indicates a higher level of social integration in distinct ways. The SE‐Political cluster engages on the field of politics (higher *z* scores) but shows less local social interaction and cultural participation. Healthy nutrition and physical activity is less common in this cluster (–.169 and –.270). The SE‐Digital chiefly socialise through digital technology and online media (.631 for friends on social networks) but also, to some degree, in the local social environment. They express a higher tendency to exercise (.439) and to engage less in the field of politics (scores between –.474 and –.709).

### Participation and healthy practices

Three clusters show high levels of participation (P) on multiple fields. The most integrated and active cluster P‐Extensive show wide ranging participation on all fields, particularly in politics and cultural consumption. They also eat healthily (.584) and tend to exercise (.582). The P‐Civically Engaged cluster stands out by a high level of voluntary engagement (2.38) and organisational memberships (1.50). Furthermore, they participate in the field of culture. They also tend to eat healthily (.549) and exercise regularly (.382) and they smoke least often compared to all other clusters (–.326). Individuals in P‐Non‐Local participate in the fields of politics and cultural consumption but attach little importance to the local social environment. They exercise regularly (.426) and they tend to eat healthily (.227) though to a lesser degree than all other clusters in this grouping. Their media use is highly skewed towards digital media (Internet use .526 compared with TV –.428).

## Life situation and formal capitals

Table 3 displays socio‐demographic and economic differences inherent in the lifestyle taxonomy. The groups differ strongly with respect to their life situation, notably education. While two thirds SD‐Physical and more than half of SD‐Extensive have either no or a lower qualification, the majority of P‐Extensive, P‐Civically Engaged and P‐Non‐Local possess either a postgraduate or higher professional degree. Individuals in the remaining groups have most often GCSE or lower. Differences in average monthly net incomes correlate with the distribution of educational qualifications. On average, P‐Extensive receive £1,533 compared to £951 for SD‐extensive.

**Table 3:** Distribution of formal social categories among lifestyle clusters

	SD‐X	SD‐L	SD‐P	SE‐P	SE‐D	P‐X	P‐CE	P‐NL
age: 16‐24	6.1	23.7	0.3	6.1	21.8	4.1	4.7	17.5
25‐44	30.2	40.9	3.9	33.5	46.3	31.6	20.4	42.1
45‐64	40.4	27.0	23.2	41.9	27.4	42.7	41.3	32.9
65 or more	23.3	8.4	72.5	18.5	4.6	21.7	33.5	7.6
gender: female	55.0	55.9	63.7	44.9	59.5	52.5	56.6	45.2
ethnicity: White British	90.2	85.4	89.5	80.9	87.7	90.3	91.9	86.1
other White	4.3	5.2	3.7	4.1	5.0	5.3	4.2	6.1
Asian	3.7	4.8	4.6	9.6	4.5	2.6	2.1	3.9
other	1.8	4.6	2.2	5.4	2.8	1.7	1.8	4.0
marital status: single	28.9	48.9	9.3	21.7	43.5	20.8	16.7	43.9
married/CP	46.1	35.1	52.4	64.9	45.0	64.4	66.9	43.8
separated or widowed	25.0	16.0	38.4	13.4	11.5	14.8	16.4	12.3
children: 1 or more	44.1	42.7	17.8	47.6	49.0	40.3	35.4	34.0
single parent	9.0	12.9	2.0	6.1	11.3	3.6	3.3	5.8
economic activity: employed	42.7	57.3	13.6	62.4	70.3	63.5	50.4	71.6
unemployed	20.3	16.6	7.1	7.5	6.9	2.9	3.2	5.6
retired	25.8	9.6	75.1	20.7	6.2	26.2	38.4	9.3
other	11.2	16.5	4.3	9.3	16.6	7.4	8.0	13.6
qualification:								
acad./professional degree	7.1	23.0	8.9	31.0	30.1	58.7	58.9	58.1
A level/GCSE	40.1	53.8	23.7	46.6	56.8	31.1	30.3	36.4
lower/other/none	52.8	23.2	67.4	22.4	13.1	10.2	10.8	5.6
NS‐SEC status:								
manager/higher prof'ional	13.5	21.1	19.2	31.6	28.6	49.5	48.2	48.2
intermediate/sm.employers	18.8	19.3	21.6	23.7	21.0	21.7	20.9	17.8
lower/routine	65.5	50.8	57.0	40.6	40.1	24.1	26.6	22.7
student/other	2.1	8.7	2.2	4.1	10.3	4.7	4.3	11.3
net income (means)	951	1,014	1,066	1,169	1,097	1,533	1,467	1,434
*statistically same as cluster*	* *	*3*	*2,5*	* *	*3*	*7*	*6,8*	*7*

**clusters in columns:** Social Disengagement ‐ Extensive (SD‐X), Local (SD‐L), Physical (SD‐P); Selective Engagement ‐ Political (SE‐P), Digital (SE‐D); Participation ‐ Extensive (P‐X), Civically Engaged (P‐CE), Non‐Local (P‐NL) · **notes:** All differences are statistically significant at p ≤ .001. Underlined figures are those that appear to distinguish a cluster particularly clearly. · For more details on socio‐demographic and economic variables in Understanding Society, see ISER 2015.

SD‐Local, P‐Non‐Local and SE‐Digital are the youngest groups with half or more being below age 35. In contrast, three quarters of SD‐Physical are above 65, which may explain their avoidance of physical and extra‐domestic activities. The share of women is also higher in this group with nearly two thirds. The most male‐dominated clusters are P‐Non‐Local and SE‐Political, the latter of which is also the most ethnically diverse.

Younger groups have a higher share of single respondents, whereas the share of married (including civil partnership) or widowed persons often reaches two thirds in older groups. The SE‐Political and SE‐Digital have most often children in their households, which, along with employment, may further restrict time budgets for participation in some fields. With 11 to 13 per cent, the share of single parents is highest in the two younger clusters SD‐Local and SE‐Digital.

## Results II: health inequalities among lifestyle clusters

### Pronounced inequalities in health and well‐being

**Figure 1:**
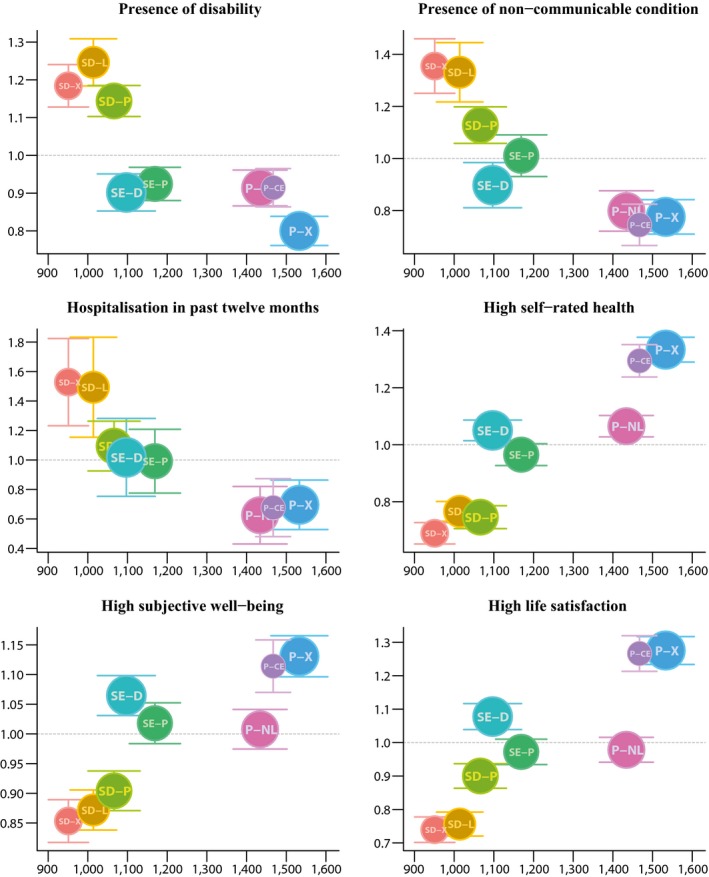
Standardised relative ratios of selected health and well‐being outcomes per lifestyle clusters. The dots are scaled to group size, and whiskers indicate 95% confidence intervals (see Table S4 for underlying figures)

The clusters exhibit pronounced inequalities on age and sex‐adjusted health indicators (Figure 1). SD‐Extensive, SD‐Local and SD‐Physical have a 1.2‐times inflated chance of disability compared to the entire sample. For SE‐Digital, disability risk is 0.9 times the average and thus significantly lower than for the demographically and economically similar SD‐Local. At the same time, this group shows similar chance of disability to the more affluent groups P‐Non‐Local and P‐Civically Engaged. Risk of disability is significantly lower for P‐Extensive with 0.8.

There is a 1.3 to 1.4 times higher prevalence of non‐communicable conditions for SD‐Extensive and SD‐Local. By contrast, for all Participation clusters the chance is 0.8 or lower. SE‐Digital indicate again significant health advantage compared to SD‐Local. Hospitalisation reflects the distribution of diagnosed conditions, although the confidence intervals are larger and, hence, the pattern is more uncertain. SD‐Extensive, SD‐Local and SD‐Physical are more likely to be hospitalised than any of the Participation clusters.

Subjective indicators of health and well‐being broadly mirror the pattern observed for objective health indicators. SD‐Extensive rate their health least often as good, while the reverse applies to P‐Extensive. P‐Civically Engaged are also significantly more likely to report good health, whereas P‐Non‐Local now reveal relative disadvantage, their probability of reporting good health is statistically identical to that of SE‐Digital. This latter cluster is again significantly better off than SD‐Local.

This pattern re‐emerges for the GHQ‐12 subjective well‐being scale. While P‐Extensive scores best, the three social disengagement clusters score worst. P‐Non‐Local indicate a similar level of subjective well‐being to SE‐Political despite their profoundly different social situation, while SE‐Digital shows similar levels of well‐being as the significantly more affluent P‐Civically Engaged. As for life satisfaction, too, P‐Extensive and P‐Civically Engaged are far more likely than average to report high life satisfaction and again contrast with SD‐Extensive and SD‐Local. P‐Non‐Local are now less satisfied than SE‐Digital.

### Lifestyle as mediator

Table 4 presents the results of the mediation analysis for disability, self‐rated health and life satisfaction. The backward step implementation of the models consistently included lifestyle categories in fully adjusted and substitution models. Age, ethnic group, education, income and social status were always selected by this method, too. The total variance accounted for is consistently the highest in fully adjusted models.

**Table 4:** Decomposition of variance accounted for by demographic, socio‐economic and lifestyle characteristics in basic, fully adjusted and substitution models

	basic		fully adjusted		substitution	
disability						
age	6.03	(5.65, 6.41)	5.34	(4.99, 5.68)	6.74	(6.31, 7.12)
sex	0.02	(0.00, 0.05)	0.02	(0.00, 0.04)	0.02	(0.00, 0.05)
marital status	0.15	(0.11, 0.20)	0.13	(0.09, 0.18)	‐	‐
children	0.60	(0.50, 0.71)	0.53	(0.43, 0.64)	‐	‐
ethnic group	0.29	(0.21, 0.37)	0.31	(0.23, 0.40)	‐	‐
qualification	1.64	(1.45, 1.83)	1.27	(1.12, 1.43)	‐	‐
income	0.20	(0.15, 0.26)	0.19	(0.14, 0.26)	‐	‐
NS‐SEC status	0.41	(0.33, 0.50)	0.35	(0.28, 0.42)	‐	‐
lifestyle cluster	‐	‐	1.66	(1.49, 1.84)	2.27	(2.06, 2.50)
total	9.36	(8.83, 9.83)	9.81	(9.26, 10.30)	9.03	(8.52, 9.49)
age+sex	6.06	(5.68, 6.43)	5.36	(5.01, 5.70)	6.76	(6.33, 7.14)
social	3.30	(3.04, 3.57)	2.79	(2.56, 3.02)	‐	‐
mediation: age+sex	‐	‐	11.48	(10.30, 12.64)	−11.53	(−14.26, −8.95)
social	‐	‐	15.50	(14.34, 16.65)	69.00	(62.50, 76.24)
degrees of freedom	30	‐	39	‐	17	‐
self‐rated health						
age	2.51	(2.27, 2.76)	2.21	(1.99, 2.43)	2.56	(2.32, 2.81)
sex	0.00	(0.00, 0.01)	0.01	(0.00, 0.02)	0.01	(0.00, 0.01)
marital status	0.13	(0.08, 0.19)	0.09	(0.05, 0.14)	‐	‐
ethnic group	0.08	(0.05, 0.13)	0.05	(0.03, 0.09)	‐	‐
qualification	2.50	(2.26, 2.74)	1.77	(1.59, 1.96)	‐	‐
income	0.57	(0.46, 0.69)	0.43	(0.34, 0.53)	‐	‐
NS‐SEC status	0.83	(0.70, 0.97)	0.65	(0.54, 0.77)	‐	‐
lifestyle cluster	‐	‐	3.07	(2.82, 3.34)	4.62	(4.28, 4.96)
total	6.62	(6.21, 7.05)	8.28	(7.82, 8.75)	7.19	(6.76, 7.63)
age+sex	2.52	(2.27, 2.76)	2.21	(1.99, 2.44)	2.57	(2.32, 2.82)
social	4.10	(3.78, 4.43)	3.00	(2.74, 3.25)	‐	‐
mediation: age+sex	‐	‐	12.05	(9.91, 14.12)	−2.17	(−5.78, 1.26)
social	‐	‐	27.03	(25.85, 28.19)	112.60	(104.07, 121.56)
degrees of freedom	28	‐	37	‐	17	‐
life satisfaction						
age	1.28	(1.10, 1.48)	1.17	(1.00, 1.35)	1.07	(0.91, 1.26)
sex	0.03	(0.01, 0.07)	0.03	(0.01, 0.06)	0.01	(0.00, 0.04)
marital status	0.88	(0.72, 1.05)	0.73	(0.59, 0.88)	‐	‐
ethnic group	0.46	(0.34, 0.58)	0.41	(0.30, 0.52)	‐	‐
qualification	0.37	(0.28, 0.47)	0.23	(0.17, 0.31)	‐	‐
income	0.71	(0.57, 0.86)	0.60	(0.48, 0.74)	‐	‐
NS‐SEC status	0.46	(0.36, 0.58)	0.39	(0.30, 0.50)	‐	‐
lifestyle cluster	‐	‐	2.56	(2.29, 2.83)	3.30	(2.99, 3.61)
total	4.19	(3.84, 4.55)	6.12	(5.70, 6.56)	4.38	(4.03, 4.76)
age+sex	1.31	(1.13, 1.51)	1.20	(1.02, 1.38)	1.09	(0.92, 1.27)
social	2.88	(2.59, 3.17)	2.37	(2.11, 2.63)	‐	‐
mediation: social	‐	‐	9.07	(6.06, 12.02)	17.21	(13.31, 21.08)
social	‐	‐	17.78	(16.23, 19.35)	114.82	(101.91, 128.96)
degrees of freedom	29	‐	38	‐	17	‐

Notes

All values in per cent (except degrees of freedom). Variance is estimated by MacFadden's R‐squared. The values in parentheses are 95% Confidence Intervals. Economic activity not significant in any of the models, children only in disability as negative association.

In the basic model of disability, age alone accounts for more than 6 per cent of the variance, whereas the role of social status variables is below 1 per cent except for qualification. The contribution of these variables reduces, however, in the fully adjusted models, to which lifestyle clusters contribute nearly 1.7 per cent. Lifestyles thus mediate 11.5 per cent of the association between age and sex and disability and 15.5 per cent of the remaining social variables. Substitution models achieve a similar fit to that of basic models. Here, the role of age and sex increases by 11.5 per cent while the remaining variance absorbed by lifestyle clusters amounts to 69 per cent of the variance accounted for by social variables in the basic models.

The statistical contributions of age and sex are lower in models of self‐rated health, whereas the social status‐related variables, notably education, gain importance. Lifestyle clusters enter the fully adjusted models with a contribution of 3 per cent, mediating 12 per cent of age and sex and 27 per cent of the remaining social variables. In substitution models, lifestyle clusters absorb 1.12 times the variance accounted for by social variables in the basic models.

The role of age and sex further recedes in life satisfaction. The variables only account for just over 1 per cent of the variance, while social variables account for nearly 3 per cent. Lifestyle clusters, contributing 2.6 per cent in fully adjusted models account for 9 per cent of the association with age and sex and 18 per cent of social variables. In substitution models, lifestyle clusters absorb 17 per cent of the variance associated with age and sex and 1.15 times the variance associated with the remaining social variables.

In summary, there is statistical evidence for a mediating role of lifestyles in the association between formal social categories and health. This evidence notwithstanding, the models should not be taken as explanatory because inasmuch as lifestyle may act as mediator, it may also be shaped by health (reverse causality). But on the whole, lifestyle clusters alone appear at least as informative as formal social categories in contextualising health, particularly when effect sizes and the lower model complexity (as reflected in degrees of freedom) of substitution models are considered.

## Discussion

### Plural lifestyles and health inequalities

The taxonomy of lifestyle clusters suggests that members of more active and socially engaged groups experience better health and well‐being. The strong prevalence of healthy practices in the participation clusters indicates that these practices are driven by the same underlying disposition that also generates high participation on other fields. Conversely, a habitus inclining towards social disengagement also finds expression in unhealthier practices associated with worse health outcomes.

Therefore, individual health‐related behaviours clearly occur neither at random nor in isolation; they are bound up in activities that correspond to the habitus that finds simultaneous expression in multiple homologous spheres of everyday life. Our ability to attribute an intrinsic, universal health effect to any given practice is thus limited. And, accordingly, health‐related practices alone cannot be decisive in accounting for group variations of health outcomes.

Within the homology of fields, heterogenous and sometimes contrasting patterns can be discerned. Social trust and support inherent in the local environment appear to be a defining characteristic of P‐Extensive, whereas P‐Non‐Local might access social capital in a wider spatial range (other cities or countries) and SE‐Digital may be more focused on online interactions. Rationalist models predicting health based on disaggregate lifestyle practices tend to ignore the different, contextual meanings and importance of practices and estimate average effects across groups with very different dispositions.

The fine‐level classification highlights further nuances in the relation between dispositions and health. Although the formal social conditions of SD‐Local and SE‐Digital are similar, the relative health advantage of the latter suggests that certain practices may moderate deeper, structural processes. Members of SE‐Digital are more socially integrated both in their local environment and in the online world. Therefore, material social circumstances need to be viewed alongside different dispositions and their aetiology to understand health and promote it. This relational interpretation of practices further permits the construction of specific contexts reflecting possible collective social experiences and shared identities, a phenomenon that has been referred to as ‘collective lifestyles’ (Frohlich *et al*. 2001). Different collective orientations may translate into differential health outcomes despite their manifestation in similar configurations of life courses and formal capitals.

The evidence from the mediation analysis supports this reasoning. Since the substitution models achieve the same or even higher explained variance than the more complex basic models, lifestyles are at least as informative as a combination of various formal social categories. Moreover, lifestyle classes characterise the practical context of individuals more explicitly than social status alone and thus permit relational and subjectivist interpretations of social difference.

### Implications for health promotion

Consequently, health promotion ought to target not the individual health‐related behaviour but consider the underlying drivers as well as their causes, all of which find expressions in many spheres of practice and indeed shape the lifestyle taxonomy itself. For example, social disengagement of SD‐Extensive may result from both material constraints and psycho‐social barriers to participation. In addition, resulting ill‐health may further restrict physical activity or social participation and thus perpetuate vicious cycles of health disadvantage and exclusion (akin to the notion of ‘reverse causality’). More fundamentally, the very existence of SD‐Extensive may itself be an expression of the workings of the political economy and its systemic ways of securing advantage for some and forcing disadvantage upon others (Scambler 2012).

Addressing the situation of SD‐Extensive would require a fully‐fledged, upstream programme at a systemic level, spanning multiple sectors. The reasoning of the psycho‐social theory of health inequalities (Wilkinson 1996) offers most pertinent suggestions, wherein the policy focus should be on social inclusion through effective redistribution, accessible education and training system alongside an accessible health care system. Isolated public health interventions are unlikely to be successful in this case.

The participation clusters present the counter‐example to social disengagement. Their capitals and dispositions seem conducive to health, and therefore, programmes focusing on individual practices according to the clusters’ detailed health profiles may be appropriate. For example, the mental health challenges of P‐Non‐Local, who are younger, have high employment rates and higher incomes, may result from lower time budgets and pressures for high personal performance in certain employment sectors. If this hypothesis can be further substantiated, employer‐focused policy initiatives and trade union engagements addressing job designs may be examples of promising preventive strategies (Layard 2013, Stansfeld *et al*. 1997). A classic public health approach may also be appropriate for P‐Extensive and P‐Civically Engaged, who show high levels of well‐being alongside inclinations towards high alcohol consumption likely in the context of socialising and leisure.

These examples illustrate how a lifestyle perspective may help identify the upstream or downstream policy domains where potential causes may be located and strategies may be formulated. In addition, consistent with Frohlich *et al*.'s (2001) idea of ‘collective lifestyles’, similar practices reflect the likelihood of shared social experiences and encounters in everyday life, which may reveal useful loci for context‐sensitive health promotion.

### Limitations

The study faces limitations from a perspective of methods and data. The cross‐sectional nature of the work restricts our ability to attribute causal relations to the patterns identified here. The main objective of this study, however, is not to explain outcomes but to identify the systematic co‐occurrence of practices across different fields (using cluster analysis) and analytically explore the extent to which these relations may enrich simpler notions of both health‐related ‘behaviours’ and the social gradient in health. The longitudinal design of the data source will offer opportunities to study causal processes as more waves become available in the future.

Cluster analysis produces ostensibly neat, distinct and consistent groups, which underplays variations among individuals of the same class through summary and cluster labelling. This abstraction is the cost of case‐based methods trading off the neglect of heterogeneities in variable‐based, analytical models on disaggregate practices. The cluster labels are therefore chosen to denote a general orientation or tendency that the underlying practices seem to describe.

The choice of the correct number of clusters is itself a debate in the clustering literature (Everitt *et al*. 2011). Here, a simple between‐group to within‐group‐variance ratio has been applied, a method that contains a subjective element and can rest on very subtle differences between cluster solutions. The eight‐cluster solution, however, offered the best statistical properties and qualitative legibility.

With respect to data, the fields could not be exhaustively represented, and, indeed, individual practices, such as smoking, cannot be unequivocally related to a single field without further information on the situation in which it is adopted. Hence, in this study, the fields constitute tools of systematisation rather than distinct and definitive domains of practice. Information on attitudes towards healthy living and health care, as used by Abel (1991), would be desirable to better identify health and the body as a field. Other practices such as the frequency of visiting friends or relatives, use of churches or other community spaces would offer a fuller representation of the diverse forms of socialising.

Although logit models are not case‐based methods, they are useful in providing evidence of the mediating role of lifestyle under the assumption that social conditions of existence remain static over the life course. Yet, it should be acknowledged that this assumption is highly simplistic and does not adequately represent the deeper structural processes that shape social differentiation.

## Concluding remarks

This paper represents a synthesis of Bourdieu's theory of social practice and methods to identify refined social groupings reflecting similar health‐relevant experience in everyday life. The approach employs Bourdieu's central concepts and principles, notably habitus, field, symbolic space and social space, but differs from his inquiry in terms of statistical methods. The chosen method – cluster analysis – permits refinement as well as consideration of health‐relevant practices and orientations shared across social groups, and it is hoped that the study may encourage wider use of case‐based methods in lieu of predictive, variable‐focused models that prompt one‐size‐fits‐all health promotion targeting the average ‘physically inactive’ or ‘materially deprived’.

Moreover, the case‐based approach contributes to the need for a realist understanding of health inequalities (Byrne 2002, Wainwright and Forbes 2000, Scambler and Scambler 2015) that permits relational interpretation (Veenstra and Burnett 2014a, 2014b) and admits a place for subjective orientations, practical logics and daily experience (Baum and Fisher 2014, Blaxter 2003). Since the taxonomy of lifestyles is itself a product of the social system with its specific ‘class and command relations’ (Scambler 2007), it broaches the question of the social root causes of health. Furthermore, as Byrne (2002) observes, a change in the taxonomy can be at least as suggestive about causal relationships than average contributions of variables to health. Therefore, although lifestyle clusters do not deliver explanations for the persistence of health inequalities with certainty, they nevertheless unveil the diverse and contingent ways in which social relations shape health.

## Supporting information


**Table S1:** Lifestyle practices and orientations in the ESRC Understanding Society survey.
**Table S2:** Missing cases on clustering variables after sample restriction.
**Table S3:** Selected sample statistics after application of longitudinal sample weights.
**Table S4:** Cluster means of age and sex‐standardised SRRs with 95% confidence intervals.
